# Development and validation of a questionnaire on nursing care for people with radiodermatitis

**DOI:** 10.1590/0034-7167-2024-0546

**Published:** 2025-12-12

**Authors:** Cristina Mara Zamarioli, Danielle Cristina Garbuio, Raquel de Souza Ramos, Emilia Campos de Carvalho

**Affiliations:** I Universidade de São Paulo. Ribeirão Preto, São Paulo, Brazil; II Centro Universitário Central Paulista. São Carlos, São Paulo, Brazil; III Instituto Nacional de Câncer. Rio de Janeiro, Rio de Janeiro, Brazil

**Keywords:** Nursing, Oncology Nursing, Nursing Process, Radiodermatitis, Nursing Care., Enfermería, Enfermería Oncológica, Proceso de Enfermería, Radiodermatitis, Atención de Enfermería.

## Abstract

**Objectives:**

to develop and validate a questionnaire on clinical practices developed by radiotherapy nurses in the process of caring for people at risk of or with radiodermatitis.

**Methods:**

methodological study, containing: establishment of the conceptual framework; definition of objectives and target population; development of items and responses; selection and organization of items; structuring of the questionnaire; content validation using the Content Validity Index; pre-testing in the target population. Data collection was performed via the Research Electronic Data Capture platform.

**Results:**

in the content validation, the questionnaire was expanded to a total of 55 questions. The overall Content Validity Index value for relevance was 0.97 and 1.00 for representativeness. The final version was considered relevant, representative, and had an appropriate layout. The target population considered the items to be relevant and clear.

**Conclusions:**

the validated questionnaire will contribute to the standardization of nursing care for people with radiodermatitis, considering current resolutions and evidence, through the application of evidence-based protocols.

## RESUMEN

**Objetivos:**

construir y validar un cuestionario elaborado por enfermeros de radioterapia sobre prácticas clínicas en el proceso de atención a personas con riesgo de radiodermatitis o padeciéndola.

**Métodos:**

estudio metodológico, conteniendo: establecimiento del marco conceptual; definición de los objetivos y de la población implicada; construcción de los ítems y respuestas; selección y organización de los ítems; estructuración del cuestionario; ratificación del contenido por el Índice de Validez de Contenido; pre-test en población objeto. Los datos se recopilaron por la plataforma *Research Electronic Data Capture*.

**Resultados:**

en la validación del contenido, el cuestionario se amplió, totalizando 55 preguntas. El valor general del Índice de Validez de Contenido para la pertinencia fue de 0,97 y para la representatividad, 1,00. La versión final se consideró relevante, representativa y con un diseño adecuado. La población destinataria consideró que los ítems eran acertados y claros.

**Conclusiones:**

el cuestionario validado contribuirá a la estandarización de los cuidados de enfermería para personas con radiodermatitis, mediante protocolos basados en las Resoluciones y axiomas vigentes.

## INTRODUCTION

The clinical practice of professional nurses in Brazil is developed in accordance with regulations governing the use of the care process, understood as the Nursing Process, to be developed at all levels and in all areas of care^(1)^. In radiotherapy services, this process can be called Nursing Consultation, due to the characteristics of the services, and it is up to nurses, in this context, to ensure the quality of care provided by nursing professionals to clients, to perform it, and to document it according to the stages of the Nursing Process^(2)^.

Considering this legal requirement, it became interesting to learn about the performance of these professionals during the provision of care in this context, especially in view of the findings in the international^(3-5)^ and national^(6)^ literature regarding the variability in the follow-up of guidelines for the prevention, assessment, and treatment of radiodermatitis. Added to this is the complexity of variables that act throughout treatment, which can contribute to the development of radiodermatitis.

Although this therapy has undergone several technological advances in the last decade, radiodermatitis is still a commonly observed side effect in clinical practice^(7,8)^, with an incidence ranging from 41% to 100%, affecting most patients undergoing radiotherapy^(9-12)^.

The literature points to the need for more robust studies, both for prevention and treatment, given that current levels of evidence are still low, as described in a review that showed that there is no standardization in most institutions for the treatment and control of radiodermatitis^(13)^, which is corroborated by other authors^(14,15)^. In general, the results of these studies indicated that the actions of professionals focus on controlling signs and symptoms when radiodermatitis has already developed, preventing infection associated with the lesion, and strategies for re-epithelialization of the affected area^(3-5,16)^. However, the literature points to a wider range of actions by nurses in cancer treatment services^(17)^.

The frequency of responses to questionnaires of this nature abroad has varied from 75% in Canada^(3)^, 81.0% in the United Kingdom^(4)^, and 25.0% in the United States of America and Europe, with the exception of the United Kingdom^(5)^. However, in Brazil, there is still no data of this nature.

It should be noted that in Brazil, a questionnaire was developed and submitted for semantic validation; it consisted of demographic and educational background data, professional training data, activities performed by the nursing professional, general guidelines on the prevention of radiodermatitis, including the presentation of clinical cases, intended for the treatment of Grade 1, 2, and 3 radiodermatitis^(18)^. However, this instrument does not cover the items or structure of the nursing care process targeted in our study, including the specification of each element of practice (diagnoses, outcomes, and interventions), the use of theoretical references in the different stages of this process, its computerization, and the use of Standardized Language Systems (SLP).

It is therefore understood that there is a gap in knowledge about how nursing care for people at risk or with radiodermatitis is configured in Brazil; therefore, it is necessary to identify how this practice is organized and being carried out. To this end, it is essential to have a valid instrument that is sensitive to current legislation.

## OBJECTIVES

To develop and validate a questionnaire on clinical practices developed by nurses in radiotherapy services in the process of caring for people at risk or in the presence of radiodermatitis.

## METHODS

### Ethical aspects

The project was submitted to and approved by the Research Ethics Committee involving Human Subjects at the institution where the research was conducted. At that time, authorization was requested to obtain informed consent forms electronically. All stages were preceded by invitations and guidance to participants.

### Study design, period, and location

**Figure 1:**
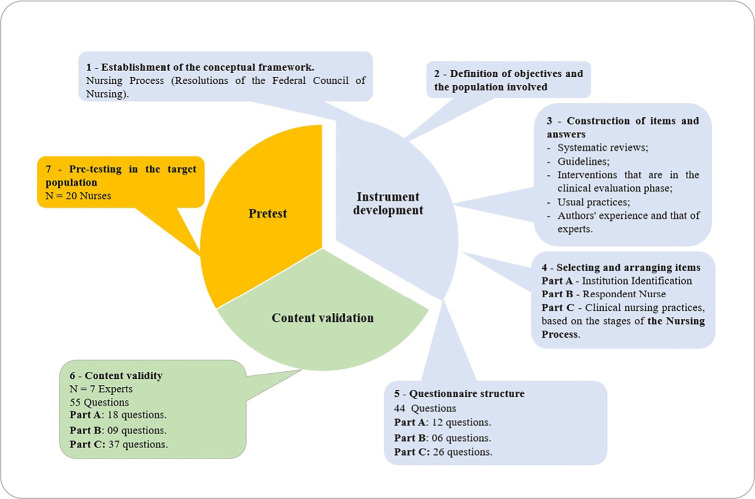
Stages of questionnaire development and validation

This is a methodological study, adopting the steps suggested in the literature^(4-24)^, illustrated in Figure 1, whose elaboration was based on the Strengthening the Reporting of Observational Studies in Epidemiology (STROBE) protocol.

The study was based at a public higher education institution in Nursing, in the countryside of the state of São Paulo. The first phase, involving the development of the instrument, was developed from July to September 2021; the second phase, involving the validation of the questionnaire, took place from February to April 2022.

The conceptual structure of the questionnaire was based on Resolution No. 358 of 2009 of the Federal Nursing Council^(20)^ in force at the time, but the discussion of the results was adjusted to the current Resolution No. 376 of 2024^(1)^. The objectives were established with the aim of understanding the nursing actions performed in different types of radiotherapy services, considering current practice and guidelines for the prevention, identification, and management of radiodermatitis^(1,6,7)^, in light of the nursing process in different care contexts^(1)^. Nurses responsible for or working in radiotherapy services, regardless of complexity, were the target population for the instrument.

The development of the items and responses of the instrument was supported by reviews available in the literature^(21-27)^; guidelines^(13,14)^; interventions that are in the clinical evaluation phase^(28,29)^, in addition to usual empirical practices; the experience of the authors and that of specialists was considered. The organization/structure of the items comprised the Identification of the Institution (A) with 6 questions, the Nurse Respondent (B) with 12 questions, and clinical nursing practices, respecting the stages of the Nursing Process (C) with 26 questions (six on Nursing Assessment, three on Nursing Diagnosis, seven on Nursing Planning, nine on Nursing Implementation, and one on Nursing Evolution). The variables contained in each part of the questionnaire are presented in the study protocol.

### Population, inclusion and exclusion criteria

For the selection of judges, criteria related to experience and qualifications in the area of interest were considered, namely: holding a doctorate or master’s degree with relevant production on the subject (radiation dermatitis, Nursing Process); having at least two years of clinical experience in radiotherapy; publishing articles in the area of interest or conducting research on the subject. This information was obtained through a survey of specialists in the aforementioned areas of knowledge by the Lattes Platform, of the National Council for Scientific and Technological Development (CNPq).

The target population of the pre-test consisted of nurses who worked, exclusively or not, in radiotherapy services registered with the Brazilian Society of Radiotherapy and who were part of a group of nurses on WhatsApp. The sample was non-probabilistic and convenience-based, after reaching the number of 20 participants.

### Study protocol

The questionnaire was sent to participants via the Research Electronic Data Capture (REDCap) platform, an electronic data capture tool hosted at Yale University^(30)^, after the principal investigator contacted them by email. To contact the target population of nurses, a direct mailing was sent via the Brazilian Society of Oncology Nursing (SBEO) and the Brazilian Society of Radiotherapy (SBRT), inviting them to participate in the research. For those who agreed to participate, a new email was sent with a link to the questionnaire, which had an estimated response time of 30 minutes and could be completed within 30 days.

The evaluation of the initial version of the questionnaire included the stages of the Nursing Process (NP)^(1,20)^ and the actions/activities inherent to each of them, considering the focus of interest in the prevention, identification, and treatment of radiodermatitis^(6,7,13,14,21-29)^.

The judges who participated in this stage evaluated the layout of the questionnaire and the relevance/representativeness of the questions and answer options, using a four-point Likert scale (ranging from 1. Not relevant or not representative to 4. Relevant and representative item). The evaluation instrument included space for comments and suggestions for item inclusion, if the expert deemed it appropriate.

The variables evaluated were: Part A - Region, State, Type of service, Length of existence (in years); Care; Approximate number of visits/day; Types of radiotherapy treatment performed at the Service; Which professional(s) perform(s) the initial assessment of the skin of the region to be irradiated; Which professional(s) perform(s) the evaluation of irradiated skin when a Grade 1 ionizing radiation-induced skin lesion appears; Which professional(s) perform(s) the evaluation of irradiated skin when a Grade 2 ionizing radiation-induced skin lesion appears; Which professional(s) perform(s) the assessment of irradiated skin when a Grade 3 ionizing radiation-induced skin lesion appears; Which professional(s) assesses irradiated skin when a Grade 4 ionizing radiation-induced skin lesion appears; Part B - Works only in the radiotherapy sector; Time since graduation (in years); Specialization in oncology; Training in nursing processes; Complementary training in ionizing radiation-induced skin lesions; Type of training; Part C - (Nursing Assessment) Indicate the patient characteristics considered in the initial assessment of the risk of developing ionizing radiation-induced skin lesions in the service; Mark the characteristics of the treatment identified in the initial assessment of the risk of developing skin lesions induced by ionizing radiation in your service; Mark the characteristics of the disease considered in the initial assessment of the risk of developing skin lesions induced by ionizing radiation in your service; How often is the patient assessed at the service? Mark the skin condition assessment tools used at the service. Mark if other skin assessment methods are used at the service. (Nursing Diagnosis) Are Nursing Diagnoses prepared at the institution? List the diagnoses or similar usually prepared. Is any classification used? What is the classification? (Nursing Planning) Are there records of goals, purpose, or expected results of nursing care? What is the usual content of these records? Are nursing prescriptions prepared? (Nursing Implementation) What information/guidance is provided to patients before the start of radiotherapy in your service? Use of topical pharmaceutical products for skin protection; Specify the duration of use in days before the start of radiotherapy; Specify the product used; The choice of product is; Indicate the information/conduct provided to the patient in case of Grade 1 ionizing radiation-induced skin injury (mild erythema and dry desquamation); Mark the information provided to the patient in case of Grade 2 ionizing radiation-induced skin damage (moderate to severe erythema, irregular moist desquamation, increased sensitivity, moderate edema); Mark the information provided to the patient in case of Grade 3 ionizing radiation-induced skin damage (confluent moist desquamation, edema); Mark the information/conduct provided to the patient in case of Grade 4 ionizing radiation-induced skin damage (ulceration, necrosis); Mark the information/conduct provided to the patient in case of post-radiotherapy treatment; Current result: What instruments are used to establish these results?; (Nursing Evolution) Performs nursing evolution; Expected results: What instruments are used to establish these results?

In the assessment carried out by nurses from the target population (pre-test), the variables clarity and relevance of the items in relation to what was intended to be investigated were considered. To assess clarity, a five-point Likert scale was applied, with 1 = Very unclear, 2 = Unclear, 3 = Clear, 4 = Very clear, and 5 = Extremely clear. To assess relevance, a five-point Likert scale was used, with 1 = Very little relevance, 2 = Little relevance, 3 = Relevant, 4 = Very relevant, and 5 = Extremely relevant. There was also space for suggestions regarding the understanding of the study’s objective, the language used, and the structure of the instrument.

### Analysis of results and statistics

The data from each stage of this project were exported directly from the REDCap platform, which generates a database in different formats, and CSV/Microsoft Excel (tables) was used for analysis in the R Software, version 4.1.2^(31)^, and Statistical Package for the Social Sciences (SPSS) software, version 25.0 (IBM).

Measures of central tendency and dispersion, frequency, and percentage were used for the characterization data of specialists and nurses. The Content Validity Index (CVI) was used in the analysis of judges’ agreement on relevance/representativeness and in the target population on clarity and pertinence. The score was calculated by summing the responses 3 and 4 for the item, divided by the total number of respondents, for relevance/representativeness, and by summing the responses 3, 4, and 5 divided by the total number of respondents, for clarity and pertinence. Items receiving 1 or 2 were reviewed. A CVI of no less than 0.78 was considered acceptable^(32)^. To assess reliability, Concordance Adjustment 1^(33)^ (CA1) was used, a more robust method that is not affected by deviations in the distribution of categories.

## RESULTS

Seven (100.0%) nurses participated in the content validation, five (71.4%) of whom were female, four (57.1%) were teachers, and three (42.9%) were nursing assistants. The average age was 44.3 years, with an average training period of 20.0 years and an average of 5.9 years of experience in radiotherapy. Two (28.6%) were teachers and specialists in the Nursing Process. Four (57.1%) had publications resulting from technological development research on products for the prevention and treatment of radiodermatitis, intervention research using such products for the prevention of radiodermatitis, as well as book chapters on care in radiotherapy treatment and radiodermatitis or on the Nursing Process.

In validating the content of the questionnaire for relevance and representativeness, the overall CVI was 0.97 and 1.00, respectively. The CVI of each question evaluated for these characteristics is presented in Table 1.

**Table 1:** Content Validity Index on the relevance and representativeness of the questions evaluated by experts (N=7), Ribeirão Preto, São Paulo, Brazil, 2023

Items	Relevance	Representativeness	Items	Relevance	Representativeness
	CVI^*^	CVI^*^		CVI^*^	CVI^*^
Q01	1.00	1.00	Q23	1.00	1.00
Q02	1.00	1.00	Q24	1.00	1.00
Q03	1.00	1.00	Q25	1.00	1.00
Q04	1.00	1.00	Q26	1.00	1.00
Q05	1.00	1.00	Q27	1.00	1.00
Q06	1.00	1.00	Q28	1.00	1.00
Q07	1.00	1.00	Q29	1.00	1.00
Q08	1.00	1.00	Q30	0.83	1.00
Q09	1.00	1.00	Q31	1.00	1.00
Q10	1.00	1.00	Q32	1.00	1.00
Q11	1.00	1.00	Q33	1.00	1.00
Q12	1.00	1.00	Q34	0.86	1.00
Q13	1.00	1.00	Q35	0.86	1.00
Q14	1.00	1.00	Q36	1.00	1.00
Q15	1.00	1.00	Q37	1.00	1.00
Q16	1.00	1.00	Q38	1.00	1.00
Q17	1.00	1.00	Q39	1.00	1.00
Q18	1.00	1.00	Q40	1.00	1.00
Q19	1.00	1.00	Q41	1.00	1.00
Q20	1.00	1.00	Q42	1.00	1.00
Q21	1.00	1.00	Q43	0.71	1.00
Q22	1.00	1.00	Q44	0.60	1.00

* *Content Validity Index.*

In the reliability analysis among evaluators on the relevance of the items, using AC1, the value was 0.9526 [0.907;0.998] (p < 0.001); however, this analysis did not show variability in terms of representativeness.

Questions 43 and 44 in part C of the questionnaire obtained an CVI lower than 0.80 and were revised (Table 1). These were questions related to the evaluation phase, current Nursing Evolution, and whether any instrument was used to establish the results. The questions were rewritten to make it clearer what was intended to be evaluated.

After evaluating the comments and suggestions of the experts, the variable city was added to part A, and 10 questions were added to part C, one on the implementation of the PE, one on the use of theoretical references, three on the use of SLP in each of the stages of the PE, four on the use of secondary coverage, and one on who implements nursing care. A questionnaire with 55 questions was then created and pre-tested on the target population.

Twenty nurses participated in the pre-test, 18 (90.0%) of whom were female, with an average professional training time of 20.2 years (standard deviation [SD] = 10.3) and 20.8 years (SD = 11.7) of professional experience, of which 9.5 years (SD = 6.1) in radiotherapy. In addition, six (30.0%) worked in radiotherapy, 12 (60.0%) had specialist qualifications (60.0%), and 13 (65.0%) had no publications in the field of radiotherapy.

It is worth noting that there was a tendency for the final questions to have a higher number of missing answers, in addition to participants answering the questions rather than simply evaluating them for relevance and clarity.

The comments pointed to a lack of understanding about the structure of the questionnaire and the reason for separating the assessment of radiodermatitis by degree. Furthermore, they considered that the questions about SLPs that were asked at different stages of the PE could be repeated.

In the assessment of the clarity and relevance of the questionnaire to the target population, the overall CVI for both was 0.9. The CVI for each question assessed for these characteristics is presented in Table 2.

**Table 2:** Content Validity Index on the clarity and relevance of the questions assessed by nurses (N=20), Ribeirão Preto, São Paulo, Brazil, 2023

Items	Clarity	Relevance	Items	Clarity	Relevance
	CVI^*^	CVI^*^		CVI^*^	CVI^*^
1	1.0	0.9	29	1.0	1.0
2	0.8	1.0	30	1.0	1.0
3	1.0	0.9	31	0.9	0.8
4	0.9	1.0	32	0.9	0.9
5	0.9	0.8	33	0.7	0.9
6	0.9	1.0	34	0.9	0.9
7	0.9	0.9	35	1.0	1.0
8	1.0	1.0	36	1.0	1.0
9	0.9	0.9	37	0.9	1.0
10	1.0	1.0	38	0.9	0.8
11	1.0	0.9	39	1.0	0.8
12	0.9	0.9	40	1.0	0.8
13	1.0	0.9	41	1.0	0.8
14	1.0	0.9	42	1.0	1.0
15	0.8	0.8	43	1.0	1.0
16	1.0	0.9	44	1.0	1.0
17	1.0	1.0	45	1.0	1.0
18	0.8	0.8	46	1.0	1.0
19	0.9	0.8	47	1.0	1.0
20	1.0	1.0	48	1.0	1.0
21	0.8	0.9	49	1.0	1.0
22	0.9	1.0	50	1.0	1.0
23	0.9	1.0	51	0.9	0.9
24	0.9	0.9	52	0.9	1.0
25	1.0	1.0	53	0.9	1.0
26	1.0	0.9	54	0.9	1.0
27	0.9	0.8	55	0.9	0.9
28	0.6	0.7			

* *Content Validity Index.*

In general, the questions were rated as very clear, clear, or somewhat clear, which was also true for relevance. The questions about the use of SLP in the Nursing Diagnosis phase had an CVI of 0.6, and the question about the content of the goals and objectives in the Nursing Planning phase had an CVI of 0.7 for clarity. As for relevance, only the question about the use of SLP in the Nursing Diagnosis phase had an CVI of 0.7. These questions were reviewed but were considered important, given the structure of the questionnaire. Regarding the item “What did you think of the layout of this questionnaire”, the average was 7.5.

## DISCUSSION

The gaps that this questionnaire sought to cover, including the specification of each element of the practice (diagnoses, results, and interventions), the use of theoretical references in the different stages of the care process, its computerization, and the use of Standardized Language Systems (SLP), were achieved.

The study participants have characteristics common to those of other studies: nurses, predominantly female^(26)^, specialists in oncology, and working in multidisciplinary teams^(34)^, with knowledge of radiotherapy and the nursing process^(1,20,35,36)^.

In this study, participants had a median age of 12.0 years of training, and the length of training can greatly influence both diagnostic and therapeutic decision-making, as these professionals tend to be experts, that is, they are able to perceive the situation as a whole based on experiences consolidated throughout their professional practice. Consequently, they can employ more decisive and objective actions and are able to predict problems and anticipate actions with extensive assertiveness^(37)^.

COFEn Resolution No. 736 of 2024 recommends basing nursing diagnoses and nursing planning on theoretical support, taking into account the client’s health status^(1)^. This is reinforced in the theoretical bases adopted in the other stages of the Nursing Process for naming the elements of practice^(38-40)^.

The nursing consultation, which must be organized and recorded according to the stages of the Nursing Process, although relevant, based on the data obtained in this study, it is noted that some stages still require attention, and this is reflected in the absence of responses or poor understanding of the issues of Nursing Planning, Implementation, and Evolution by the participants. This finding deserves to be further explored in services and throughout patient follow-up.

The literature shows that the clinical evaluation of patients who will undergo radiotherapy can contribute to the prevention, treatment, and standardization of language for the development of care protocols^(41)^ and needs to be instituted in services, considering the risk of developing radiodermatitis in each patient on an individualized basis. Nursing consultations are important in minimizing severity and reducing treatment interruption^(42-45)^; however, they need to be effectively implemented in services and throughout patient follow-up.

This questionnaire pointed out alternatives for usual conduct/prescriptions, recommended both nationally and internationally^(6,7,13,14,46-48)^, some of which are more general and others directed at different degrees of radiodermatitis.

### Study limitations

The limitations of this study are related to the low number of responses to the final questions of the questionnaire by nurses, which may have different explanations; one of them related to the size of the questionnaire; another to its structure, which highlighted the phases of the NP, making them visible and hindering the understanding of a topic that still needs to be further explored; another to the real difficulty of preventing and treating radiodermatitis in its different degrees.

Some of them are very useful as an opportunity to describe the need for future research on radiodermatitis, on the Nursing Process and its difficulty of operationalization, especially in the later phases, on the use of measurement instruments in the clinical evaluation of patients, on the use of SLP in the different phases of the NP, among others.

### Contributions to the field of work

The validated instrument will enable diagnosis of practice in the sector, aspects relevant to the discussion on the training of the nursing team in radiotherapy, considering the difficulties and potentialities of applying the PE in clinical practice.

Furthermore, it strengthens the scientific basis for standardizing care in radiodermatitis, which may influence care protocols and professional training.

## CONCLUSIONS

The questionnaire was constructed following rigorous methodology, comprehensively covering issues that are known and need to be investigated by professionals at radiotherapy centers, as well as including each of these aspects in the stages of the NP, to make it visible. The final version was considered relevant, representative, and presented an appropriate layout. The target population considered the items to be relevant and clear.

The study also contributes to the discussion of the need for investment in training and assistance for the operationalization of the Nursing Process, the implementation of some stages, notably those that are the exclusive competence of the nursing professional.

## Data Availability

The research data are available within the article.
