# Supplementary material for: Development and validation of a questionnaire on nursing care for people with radiodermatitis
Source: Rev Bras Enferm. 2025 Dec 12;78(Suppl 4):e20240546. doi: 10.1590/0034-7167-2024-0546 (PMC12707760; doi:10.1590/0034-7167-2024-0546)
Supplement: Translation

# Translation: Construção e validação de questionário sobre cuidados de Enfermagem à pessoa com radiodermatite

**DOI:** 10.1590/0034-7167-2024-0546pt

**Authors:** Cristina Mara Zamarioli, Danielle Cristina Garbuio, Raquel de Souza Ramos, Emilia Campos de Carvalho

**Affiliations:** I Universidade de São Paulo. Ribeirão Preto, São Paulo, Brasil; II Centro Universitário Central Paulista. São Carlos, São Paulo, Brasil; III Instituto Nacional de Câncer. Rio de Janeiro, Rio de Janeiro, Brasil

**Keywords:** Enfermagem, Enfermagem Oncológica, Processo de Enfermagem, Radiodermatite, Cuidados de Enfermagem.

## Abstract

**Objetivos:**

construir e validar um questionário sobre práticas clínicas desenvolvidas por enfermeiros de radioterapia no processo de cuidar da pessoa com risco ou presença de radiodermatites.

**Métodos:**

estudo metodológico, contendo: estabelecimento da estrutura conceitual; definição dos objetivos e da população envolvida; construção dos itens e das respostas; seleção e organização dos itens; estruturação do questionário; validação de conteúdo pelo Índice de Validade de Conteúdo; pré-teste na população-alvo. A coleta foi realizada via plataforma *Research Electronic Data Capture*.

**Resultados:**

na validação de conteúdo o questionário foi ampliado totalizando 55 questões. O valor do Índice de Validade de Conteúdo geral quanto à relevância foi de 0,97 e, de 1,00 para a representatividade. A versão final foi considerada relevante, representativa e apresentou *layout* adequado. A população-alvo considerou os itens pertinentes e claros.

**Conclusões:**

o questionário validado contribuirá para a padronização do cuidado de enfermagem à pessoa com radiodermatite, à luz das Resoluções e evidências vigentes, por meio de aplicação de protocolos baseados em evidências.

## INTRODUÇÃO

A prática clínica do profissional enfermeiro no Brasil é desenvolvida atendendo-se à regulamentação do uso do processo de cuidar, entendido como Processo de Enfermagem, a ser desenvolvido em todos os níveis e âmbitos da assistência^(1)^. Nos serviços de radioterapia tal processo pode ser chamado de Consulta de Enfermagem, pelas características dos serviços, cabendo aos enfermeiros, neste contexto, assegurar a qualidade da assistência prestada pelos profissionais de Enfermagem aos clientes, executá-la e documentá-la conforme as etapas do Processo de Enfermagem^(2)^.

Considerando esta exigência legal, tornou-se interesse conhecer a atuação destes profissionais durante a prestação do cuidado nesse contexto, em especial, frente aos achados da literatura internacional^(3-5)^ e nacional^(6)^ quanto à variabilidade do seguimento das diretrizes para a prevenção, avaliação e tratamento das radiodermatites. Somam-se, ainda, a complexidade de variáveis que atuam ao longo do tratamento, que podem contribuir para o desenvolvimento da radiodermatite.

Embora essa terapêutica tenha passado por diversos avanços tecnológicos na última década, a radiodermatite ainda é um efeito colateral comumente observado na prática clínica^(7,8)^, uma incidência que varia entre 41% e 100%, acometendo a maior parte dos pacientes submetidos à radioterapia^(9-12)^.

A literatura aponta a necessidade de realização de estudos mais robustos, tanto para a prevenção quanto para o seu tratamento, em função dos níveis de evidência atuais que ainda são baixos, como descrito em revisão que evidenciou não haver padronização, na maioria das instituições, para o tratamento e controle das radiodermatites^(13)^, tal dado foi corroborado por outros autores^(14,15)^. De forma geral, os resultados desses estudos apontaram que as ações dos profissionais se concentram no controle de sinais e sintomas quando a radiodermatite já está desenvolvida, na prevenção de infecção associada à lesão e nas estratégias para reepitelização da área afetada^(3-5,16)^. Todavia, a literatura aponta ser maior o leque de ações do enfermeiro dos serviços de tratamento oncológico^(17)^.

A frequência de respostas aos questionários dessa natureza no exterior tem variado de 75% no Canadá^(3)^, 81,0% no Reino Unido^(4)^ e 25,0% nos Estados Unidos da América e na Europa, com exceção do Reino Unido^(5)^. Todavia, no Brasil, ainda não há dados desta natureza.

Destaca-se que no Brasil um questionário foi desenvolvido e submetido à validação semântica; composto por dados demográficos e formação educacional, dados de formação profissional, atividades realizadas pelo profissional enfermeiro, orientações gerais acerca da prevenção de radiodermatites, incluindo a apresentação de casos clínicos, destinadas ao tratamento de radiodermatites de Graus 1, 2 e 3^(18)^. Entretanto, tal instrumento não contempla os itens ou a estrutura do processo de cuidar em Enfermagem almejados em nosso estudo, entre eles, cita-se a especificação de cada elemento da prática (diagnósticos, resultados e intervenções), o uso de referenciais teóricos nas diferentes etapas do processo, informatização, além do uso de Sistemas de Linguagem Padronizada (SLP).

Entende-se, portanto, que existe uma lacuna no conhecimento sobre como se configura, no território nacional, o cuidado de enfermagem para pessoas na situação de risco ou presença de radiodermite; portanto, torna-se necessário identificar como tal prática está organizada e está sendo realizada. Para tanto, é fundamental que haja um instrumento válido e sensível à legislação vigente.

## OBJETIVOS

Construir e validar um questionário sobre práticas clínicas desenvolvidas por enfermeiros nos serviços de radioterapia no processo de cuidar da pessoa com risco ou na presença de radiodermatites.

## MÉTODOS

### Aspectos éticos

O projeto foi encaminhado e aprovado pelo Comitê de Ética em Pesquisa envolvendo seres humanos da Instituição sede da pesquisa. Na ocasião, solicitou-se a autorização para acolhimento dos Termos de Consentimento Livre e Esclarecidos (TCLE) via eletrônica. Todas as etapas foram precedidas de convite e orientações aos participantes.

### Desenho, período e local do estudo

Trata-se de um estudo metodológico, adotando-se as etapas sugeridas na literatura^(4-24)^, ilustradas na Figura 1, cuja elaboração apoiou-se no protocolo *Strengthening the Reporting of Observational Studies in Epidemiology* (STROBE).

**Figura 1:** Etapas da construção e validação do questionário

O estudo foi sediado em uma instituição pública de ensino superior em Enfermagem, do interior do estado de São Paulo. A primeira fase, de construção do instrumento, foi desenvolvida de julho a setembro de 2021; a segunda, relativa à validação do questionário, de fevereiro a abril de 2022.

A estrutura conceitual do questionário apoiou-se na Resolução n° 358 de 2009 do Conselho Federal de Enfermagem^(20)^ vigente à época, porém a discussão dos resultados foi ajustada à atual Resolução n° 376 de 2024^(1)^. O estabelecimento dos objetivos apoiou-se no propósito de conhecer as ações de Enfermagem executadas nos diferentes tipos de serviços de radioterapia, considerando-se a prática vigente e as diretrizes para a prevenção, identificação e manejo de radiodermatite^(1,6,7)^, à luz do Processo de Enfermagem nos distintos contextos do cuidado^(1)^. Enfermeiros responsáveis ou que atuavam nos serviços de radioterapia, independentemente da complexidade, foram a população de interesse para o instrumento.

A construção dos itens e das respostas do instrumento foram apoiadas em revisões disponíveis na literatura^(21-27)^; *guidelines* ^(13,14)^; de intervenções que estão em fase de avaliação clínica^(28,29)^, além das práticas usuais empíricas; a experiência das autoras e a de especialistas foram consideradas. A organização/estruturação dos itens compreendeu a Identificação da Instituição (A) com 6 questões, o Respondente Enfermeiro (B) com 12 questões e as práticas clínicas de Enfermagem, respeitando-se as etapas do Processo de Enfermagem (C) com 26 questões (seis sobre Avaliação de Enfermagem, três sobre Diagnóstico de Enfermagem, sete sobre Planejamento de Enfermagem, nove sobre Implementação de Enfermagem e uma sobre Evolução de Enfermagem). As variáveis contidas em cada parte do questionário estão apresentadas no protocolo do estudo.

### População, critérios de inclusão e exclusão

Foram considerados, para a seleção dos juízes, quesitos referentes à experiência e a qualificação na área de interesse, sendo eles: possuir título de doutor ou mestre com produção relevante sobre a temática (radiodermatite, Processo de Enfermagem); ter experiência clínica de pelo menos dois anos em radioterapia; publicar artigos na área de interesse ou pesquisar sobre o tema. As informações foram obtidas através de um levantamento sobre os especialistas nas referidas áreas do conhecimento pela Plataforma Lattes, do Conselho Nacional de Desenvolvimento Científico e Tecnológico (CNPq).

A população-alvo do pré-teste foi composta por enfermeiros que atuavam, exclusivamente ou não, nos serviços de radioterapia cadastrados na Sociedade Brasileira de Radioterapia do país e que faziam parte de um grupo de enfermeiros no WhatsApp. A amostra foi do tipo não probabilística e por conveniência, após atingir o número de 20.

### Protocolo do estudo

O questionário foi encaminhado aos participantes via plataforma *Research Electronic Data Capture* (REDCap), isto é, ferramentas de captura eletrônica de dados hospedadas na Universidade de Yale^(30)^, após a pesquisadora principal entrar em contato, por e-mail. Para contato com os enfermeiros população-alvo, utilizou-se a mala direta via Sociedade Brasileira de Enfermagem Oncológica (SBEO) e Sociedade Brasileira de Radioterapia (SBRT), realizando o convite para participação na pesquisa. Para aqueles que aceitaram participar, foi enviado um novo e-mail com o *link* para avaliação do questionário, que tinha um tempo previsto para resposta de 30 minutos, podendo ser respondido em até de 30 dias.

A avaliação da versão inicial do questionário teve como conteúdo as etapas do Processo de Enfermagem (PE)^(1,20)^ e as ações/atividades inerentes a cada uma delas, considerando o foco de interesse em prevenção, identificação e tratamento de radiodermatite^(6,7,13,14,21-29)^.

Os juízes que participaram desta etapa avaliaram o *layout* do questionário e, ainda, a relevância/representatividade das questões e alternativas de respostas, utilizando uma escala do tipo *Likert* de quatro pontos (variando de 1) Não relevante ou não representativo a 4) Item relevante e representativo). No instrumento de avaliação havia espaço para comentários e sugestão de inclusão de item, caso o especialista julgasse apropriado.

As variáveis avaliadas foram: Parte A - Região, Estado, Tipo de serviço, Tempo de existência (em anos); Atendimento; Número aproximado de atendimentos/dia; Tipos de tratamento radioterápico realizados no Serviço; Qual(is) profissional(ais) efetua(m) a avaliação inicial da pele da região a ser irradiada; Qual(is) profissional(ais) efetua(m) a avaliação da pele irradiada quando surge lesão cutânea induzida por radiação ionizante Grau 1; Qual(is) profissional(ais) efetua(m) a avaliação da pele irradiada quando surge lesão cutânea induzida por radiação ionizante Grau 2; Qual(is) profissional(ais) efetua(m) a avaliação da pele irradiada quando surge lesão cutânea induzida por radiação ionizante Grau 3; Qual(is) profissional(ais) efetua(m) a avaliação da pele irradiada quando surge lesão cutânea induzida por radiação ionizante Grau 4; Parte B - Atua apenas no setor de radioterapia; Tempo de formado (em anos); Possui especialização em oncologia; Possui formação sobre processo de enfermagem; Possui formação complementar sobre lesão cutânea induzida por radiação ionizante; Qual formação; Parte C - (Avaliação de Enfermagem) Assinale as características do paciente consideradas na avaliação inicial de risco de desenvolvimento de lesão cutânea induzida por radiação ionizante no serviço; Assinale as características do tratamento identificadas na avaliação inicial de risco de desenvolvimento de lesão cutânea induzida por radiação ionizante no seu serviço; Assinale as características da doença consideradas na avaliação inicial de risco de desenvolvimento de lesão cutânea induzida por radiação ionizante no seu serviço; Qual a frequência de avaliação do paciente no serviço; Assinale os instrumentos de avaliação da condição da pele utilizados no serviço; Assinale se outros métodos de avaliação da pele são utilizados no serviço; (Diagnóstico de Enfermagem) São elaborados Diagnósticos de Enfermagem na instituição; Cite os diagnósticos ou similares usualmente elaborados; É utilizada alguma classificação; Qual a classificação; (Planejamento de Enfermagem) Há registro de metas, propósito ou resultados esperados da assistência de enfermagem; Qual o conteúdo usual desse registro; Há elaboração de prescrição de enfermagem; (Implementação de Enfermagem) Quais as informações/condutas fornecidas ao paciente antes do início da radioterapia no seu serviço; Uso de produto farmacêutico tópico para proteção da pele; Especificar tempo de uso em dias antes do início da radioterapia; Especificar o produto usado; A escolha do produto é; Assinale as informações/condutas fornecidas ao paciente em caso de lesão cutânea induzida por radiação ionizante Grau 1 (Eritema fraco e descamação seca); Assinale as informações fornecidas ao paciente em caso de lesão cutânea induzida por radiação ionizante Grau 2 (Eritema moderado a intenso, descamação úmida irregular, aumento da sensibilidade, edema moderado); Assinale as informações fornecidas ao paciente em caso de lesão cutânea induzida por radiação ionizante Grau 3 (Descamação úmida confluente, edema); Assinale as informações/condutas fornecidas ao paciente em caso de lesão cutânea induzida por radiação ionizante Grau 4 (Ulceração, necrose); Assinale as informações/condutas fornecidas ao paciente em caso de pós-tratamento radioterápico; Resultado atual: Quais instrumentos são utilizados para estabelecer estes resultados?; (Evolução de Enfermagem) Realiza a evolução de Enfermagem; Resultados esperados: Quais instrumentos são utilizados para estabelecer estes resultados?.

Na avaliação feita por enfermeiros da população-alvo (pré-teste), considerou-se as variáveis clareza e pertinência dos itens em relação ao que se pretendia investigar. Para a avaliar a clareza foi aplicada uma escala do tipo *Likert* de cinco pontos, sendo 1. Pouquíssima clareza, 2. Pouca clareza, 3. Clara, 4. Muita clareza e 5. Muitíssima clareza. Já para avaliar a pertinência foi utilizada uma escala do tipo *Likert* de cinco pontos, sendo 1. Pouquíssima pertinência, 2. Pouca pertinência, 3. Pertinente, 4. Muita pertinência e 5. Muitíssima pertinência. Ainda, havia espaço para sugestões abordando o entendimento do objetivo do estudo, da linguagem empregada e a estrutura do instrumento.

### Análise dos resultados e estatística

Os dados de cada etapa deste projeto foram exportados diretamente da plataforma REDCap, que gera um banco em diferentes extensões e a utilizada foi CSV/Microsoft Excel (*tabels*), para análise no Programa R, versão 4.1.2^(31)^ e *Software Statistical Package for the Social Sciences* (SPSS), versão 25.0 (IBM).

Foram utilizadas medidas de tendência central e de dispersão, frequência e porcentagem para os dados de caracterização dos especialistas e dos enfermeiros. Na análise de concordância dos juízes sobre relevância/representatividade e na população-alvo sobre clareza e pertinência, utilizou-se o Índice de Validade de Conteúdo (IVC). O escore foi calculado pela soma das respostas 3 e 4 para o item, dividido pelo número total de respondentes, para relevância/representatividade e, pela soma das respostas 3, 4 e 5 dividido pelo número total de respondentes, para clareza e pertinência. Os itens que receberem 1 ou 2 foram revistos. Foi considerado aceito o IVC não inferior a 0,78^(32)^. Para avaliar a confiabilidade, utilizou-se o Ajuste de Concordância 1^(33)^ (AC1), um método mais robusto, que não é afetado por desvios na distribuição das categorias.

## RESULTADOS

Participaram da validação de conteúdo sete (100,0%) enfermeiros, sendo cinco (71,4%) do sexo feminino, quatro (57,1%) docentes e três (42,9%) enfermeiros assistenciais. A média etária foi de 44,3 anos, tempo de formação médio de 20,0 anos, média de tempo de atuação na radioterapia de 5,9 anos. Dois (28,6%) eram docentes e especialistas em Processo de Enfermagem. Quatro (57,1%) tinham publicação oriundas de pesquisas de desenvolvimento tecnológico de produtos para prevenção e tratamento de radiodermatites, pesquisas de intervenção utilizando tais produtos para prevenção de radiodermatites, além de capítulos de livros sobre cuidados frente ao tratamento radioterápico e radiodermatite ou sobre Processo de Enfermagem.

Na validação do conteúdo do questionário quanto à relevância e representatividade, o IVC geral foi de 0,97 e de 1,00, respectivamente. O IVC de cada questão avaliada quanto a estas características está apresentado na Tabela 1.

**Tabela 1:** Índice de Validade de Conteúdo sobre a relevância e representatividade das questões avaliados pelos peritos (N=7), Ribeirão Preto, São Paulo, Brasil, 2023

Itens	Relevância	Representatividade	Itens	Relevância	Representatividade
	IVC^*^	IVC^*^		IVC^*^	IVC^*^
Q01	1,00	1,00	Q23	1,00	1,00
Q02	1,00	1,00	Q24	1,00	1,00
Q03	1,00	1,00	Q25	1,00	1,00
Q04	1,00	1,00	Q26	1,00	1,00
Q05	1,00	1,00	Q27	1,00	1,00
Q06	1,00	1,00	Q28	1,00	1,00
Q07	1,00	1,00	Q29	1,00	1,00
Q08	1,00	1,00	Q30	0,83	1,00
Q09	1,00	1,00	Q31	1,00	1,00
Q10	1,00	1,00	Q32	1,00	1,00
Q11	1,00	1,00	Q33	1,00	1,00
Q12	1,00	1,00	Q34	0,86	1,00
Q13	1,00	1,00	Q35	0,86	1,00
Q14	1,00	1,00	Q36	1,00	1,00
Q15	1,00	1,00	Q37	1,00	1,00
Q16	1,00	1,00	Q38	1,00	1,00
Q17	1,00	1,00	Q39	1,00	1,00
Q18	1,00	1,00	Q40	1,00	1,00
Q19	1,00	1,00	Q41	1,00	1,00
Q20	1,00	1,00	Q42	1,00	1,00
Q21	1,00	1,00	Q43	0,71	1,00
Q22	1,00	1,00	Q44	0,60	1,00

* *Índice de Validade de Conteúdo.*

Na análise de confiabilidade entre os avaliadores sobre a relevância dos itens como um todo, pelo AC1, foi valor de 0,9526 [0,907;0,998] (p < 0,001); porém esta análise não teve variabilidade quanto à representatividade.

As questões 43 e 44 da parte C do questionário obtiveram IVC menor que 0,80 e foram revistas (Tabela 1). Tratavam-se de questões relacionadas à fase de avaliação, atual Evolução de Enfermagem, e se era utilizado algum instrumento para estabelecer os resultados. As questões foram reescritas para deixar mais claro o que se pretendia avaliar.

Após a avaliação dos comentários e sugestões dos especialistas, na parte A foi acrescentada a variável cidade e na Parte C, 10 questões, sendo uma sobre realização do PE, uma para utilização de referencial teórico, três sobre utilização de SLP em cada uma das etapas do PE, quatro sobre uso de coberturas secundárias, uma sobre quem implementa a assistência de enfermagem. Confeccionou-se, então, um questionário com 55 questões, que foi submetido ao pré-teste na população-alvo.

Participaram do pré-teste 20 enfermeiros, sendo 18 (90,0%) do sexo feminino, com tempo médio de formação profissional de 20,2 anos (desvio-padrão [DP] =10,3) e com 20,8 anos (DP=11,7) de atuação profissional, dos quais 9,5 anos (DP=6,1) na radioterapia. Ainda, seis (30,0%) atuavam na radioterapia, 12 (60,0%) possuíam o título de especialista (60,0%) e 13 (65,0%) não possuíam publicação na área de radioterapia.

Cabe comentar que houve uma tendência nas questões finais de haver maior número de ausências de respostas, além dos participantes responderem às questões e não somente avaliá-las quanto à pertinência e clareza.

Os comentários apontaram para a falta de compreensão acerca da estrutura do questionário, o porquê da separação da avaliação da radiodermite por grau. Ainda, consideraram que as questões acerca dos SLP, que foram feitas em diferentes fases do PE, poderiam estar repetidas.

Na avaliação da clareza e da pertinência do questionário com a população-alvo, o IVC geral de ambas foi de 0,9. O IVC de cada questão avaliada quanto às características está apresentado na Tabela 2.

**Tabela 2:** Índice de Validade de Conteúdo sobre a clareza e pertinência das questões avaliadas pelos enfermeiros (N=20), Ribeirão Preto, São Paulo, Brasil, 2023

Itens	Clareza	Pertinência	Itens	Clareza	Pertinência
	IVC^*^	IVC^*^		IVC^*^	IVC^*^
1	1,0	0,9	29	1,0	1,0
2	0,8	1,0	30	1,0	1,0
3	1,0	0,9	31	0,9	0,8
4	0,9	1,0	32	0,9	0,9
5	0,9	0,8	33	0,7	0,9
6	0,9	1,0	34	0,9	0,9
7	0,9	0,9	35	1,0	1,0
8	1,0	1,0	36	1,0	1,0
9	0,9	0,9	37	0,9	1,0
10	1,0	1,0	38	0,9	0,8
11	1,0	0,9	39	1,0	0,8
12	0,9	0,9	40	1,0	0,8
13	1,0	0,9	41	1,0	0,8
14	1,0	0,9	42	1,0	1,0
15	0,8	0,8	43	1,0	1,0
16	1,0	0,9	44	1,0	1,0
17	1,0	1,0	45	1,0	1,0
18	0,8	0,8	46	1,0	1,0
19	0,9	0,8	47	1,0	1,0
20	1,0	1,0	48	1,0	1,0
21	0,8	0,9	49	1,0	1,0
22	0,9	1,0	50	1,0	1,0
23	0,9	1,0	51	0,9	0,9
24	0,9	0,9	52	0,9	1,0
25	1,0	1,0	53	0,9	1,0
26	1,0	0,9	54	0,9	1,0
27	0,9	0,8	55	0,9	0,9
28	0,6	0,7			

* *Índice de Validade de Conteúdo.*

De um modo geral, as questões foram avaliadas como muitíssimo claras, muito claras ou claras, o que se reproduziu para a pertinência. As questões sobre utilização de SLP na fase de Diagnóstico de Enfermagem teve IVC de 0,6 e a que questionava o conteúdo das metas e objetivos na fase do Planejamento de Enfermagem de 0,7 para clareza. E, quanto à pertinência, somente a questão sobre utilização de SLP na fase de Diagnóstico de Enfermagem teve IVC de 0,7. Tais questões foram revistas, mas foram consideradas importantes, considerando a estrutura do questionário. Quanto ao item “O que você achou do layout desse questionário”, a média foi de 7,5.

## DISCUSSÃO

As lacunas que este questionário buscou cobrir - entre elas, a especificação de cada elemento da prática (diagnósticos, resultados e intervenções), o uso de referenciais teóricos nas diferentes etapas do processo de cuidar, informatização do mesmo, além do uso de Sistemas de Linguagem Padronizada (SLP) - foram alcançadas.

Os participantes do estudo apresentam características comuns às de outros estudos: enfermeiros, predominantemente do sexo feminino^(26)^, especialistas em oncologia e que atuam em equipes multiprofissionais^(34)^, com conhecimento sobre radioterapia e Processo de Enfermagem^(1,20,35,36)^.

Neste estudo, os participantes tinham uma mediana de idade de 12,0 anos de formação e o tempo de formação pode influenciar, sobremaneira, na tomada de decisão, tanto diagnóstica quanto terapêutica, à medida que o(a)s profissionais tendem a ser *experts*, ou seja, conseguem perceber a situação como um todo a partir de vivências consolidadas ao longo da prática profissional. Consequentemente, podem empregar ações mais resolutivas e objetivas e são capazes de prever problemas e antecipar ações com extensa assertividade^(37)^.

A Resolução do COFEn n° 736 de 2024 aponta a recomendação de embasamento em algum suporte teórico, estabelecimento de Diagnósticos de Enfermagem, Planejamento de Enfermagem, em face do estado de saúde do cliente^(1)^. Isso se reforça nas bases teóricas adotadas nas demais etapas do Processo de Enfermagem, para nomeação dos elementos da prática^(38-40)^.

A consulta de Enfermagem, que deve ser organizada e registrada conforme as etapas do Processo de Enfermagem, embora relevante, a partir dos dados obtidos neste estudo, nota-se que algumas etapas ainda são motivo de atenção, e o reflexo disso é a ausência de respostas ou pouca compreensão acerca das questões sobre Planejamento, Implementação e Evolução de Enfermagem pelos participantes. Tal achado merece ser melhor explorado nos serviços e durante todo o acompanhamento do paciente.

A literatura traz que a avaliação clínica do paciente que será submetido à radioterapia pode contribuir para a prevenção, tratamento e padronização de linguagem para a construção de protocolos assistenciais^(41)^ e precisa ser instituída nos serviços, considerando o risco de desenvolvimento das radiodermatites, em cada paciente, de forma individualizada. E, que a consulta de Enfermagem é importante no tocante à minimização de severidade e diminuição da interrupção do tratamento^(42-45)^; porém, precisa ser viabilizada de forma efetiva nos serviços, e durante todo o acompanhamento do paciente.

O presente questionário apontou alternativas de condutas/prescrições usuais, preconizadas tanto nacional quanto internacionalmente^(6,7,13,14,46-48)^, sendo algumas mais gerais e outras direcionadas para os diferentes graus da radiodermatite.

### Limitações do estudo

As limitações desse estudo estão relacionadas ao número reduzido de respostas às questões finais do questionário pelos enfermeiros, o que pode ter diferentes explicações; uma delas relacionada ao tamanho do questionário; outra à sua estrutura, que evidenciou as fases do PE tornando-as visíveis e dificultando a compreensão de um tema que ainda precisa ser melhor explorado; outra à real dificuldade de ser prevenir e tratar as radiodermatites nos seus diferentes graus.

Algumas delas são muito úteis como uma oportunidade para descrever as necessidades de pesquisas futuras sobre radiodermatites, sobre o Processo de Enfermagem e a sua dificuldade de operacionalização, principalmente das últimas fases, à utilização de instrumentos de medidas na avaliação clínica dos pacientes, à utilização de SLP nas diferentes fases do PE, entre outras.

### Contribuições para a Área

O instrumento validado permitirá diagnóstico da prática no setor, aspectos relevantes para a discussão sobre a formação da equipe de enfermagem em radioterapia, considerando as dificuldades e potencialidades da aplicação do PE na prática clínica.

Ainda, fortalece-se a base científica para a padronização do cuidado em radiodermatite, o que pode influenciar protocolos assistenciais e formação profissional.

## CONCLUSÕES

A construção do questionário seguiu um rigor metodológico, contemplando, de forma abrangente, questões que são conhecidas e que precisam ser investigadas pelos profissionais dos centros de radioterapia, bem como a inclusão de cada um desses aspectos nas etapas do PE, tentando torná-lo visível. A versão final foi considerada relevante, representativa e apresentou *layout* adequado. A população-alvo considerou os itens pertinentes e claros.

O estudo contribui, ainda, para a discussão da necessidade de investimento no treinamento e auxílio para a operacionalização do Processo de Enfermagem, a realização de algumas etapas, notadamente as que são de competência exclusiva do profissional enfermeiro.

## Data Availability

Os dados da pesquisa estão disponíveis no corpo do artigo.
